# Construction and empirical analysis of a quantitative model on the relationship between budget control and financial performance in management accounting—Evidence from Russian enterprises

**DOI:** 10.1371/journal.pone.0337863

**Published:** 2026-07-15

**Authors:** Ning Tie

**Affiliations:** School of Economics and Management of Anyang University, Anyang, Henan, China; Instituto Tecnologico Autonomo de Mexico, MEXICO

## Abstract

Currently, many enterprises face issues in budget management, such as superficial budgeting, lax execution, and insufficient feedback, leaving the mechanism linking budgetary control and financial performance unclear. Drawing on management accounting and financial management theories, this study constructs a research model encompassing direct, mediating, and moderating effects and conducts a quantitative empirical analysis using Russian enterprises as the research sample. Empirical analyses are conducted using both publicly available data from the Russian Financial Statements Database (RFSD) (https://github.com/irlcode/RFSD) and the collected survey data. In terms of variable measurement, budget control is evaluated across three dimensions—budget formulation, execution, and feedback—with mean scores ranging from 3.7 to 3.9, indicating that most enterprises place considerable emphasis on the establishment of budgeting systems. The mean financial performance score ranges from 3.5 to 3.6, suggesting a moderately favorable performance level. Empirical results reveal a significant positive correlation between budget control and financial performance (r > 0.5). In the regression model, the coefficient of budget control is significantly positive, and the explanatory power of the model increases from 0.183 to 0.361 after incorporating budget control, confirming its direct contribution to performance improvement. Further mediation analysis indicates that resource allocation efficiency and internal management processes serve as significant intermediaries between budget control and financial performance, with indirect effects accounting for 29.4% of the total effect. This suggests that budget control primarily enhances performance indirectly by improving internal management mechanisms. The moderation analysis shows that, within the Russian institutional environment, firm size and governance structure strengthen the positive impact of budget control, whereas external environmental uncertainty weakens it. These findings provide empirical evidence for understanding the performance effects of budget control under specific institutional and economic contexts and offer practical guidance for optimizing budget management in Russian enterprises and other transitional economies.

## Introduction

In modern enterprise management, financial performance is a key indicator of operational outcomes and a crucial determinant of sustainable development. With intensifying market competition and an ever-changing resource allocation environment, enterprises are placing increasing emphasis on financial performance [1,2]. However, the mechanisms underlying financial performance are not unidimensional; they are shaped by multiple interacting factors, including strategic orientation, management accounting practices, organizational governance, and the external environment [3]. Among these, budget control, as a core component of management accounting, is widely regarded as the critical link between resource allocation and performance output. Budget control provides a quantitative foundation for setting strategic objectives and operational plans, while also enhancing management efficiency and optimizing resource utilization through mechanisms of constraint and incentive [4,5]. At the theoretical level, budget control—through its cyclical process of planning, execution, and feedback—helps reduce information asymmetry and strengthen internal control. In practice, sound budget management improves fund utilization efficiency, operational results, and return on capital. Nevertheless, in reality, many enterprises treat budgeting as a mere formality, fail to execute it effectively, or neglect performance feedback, making the relationship between budget control and financial performance both complex and controversial.

Against this backdrop, this study constructs a quantitative research model based on management accounting theory to examine the relationship between budget control and financial performance using empirical data. Through descriptive analysis, correlation testing, regression modeling, and examinations of moderating and mediating effects, the study systematically explores both the direct and indirect mechanisms through which budget control influences financial performance. The study aims to address the current gap in quantitative empirical studies, while offering enterprises scientific and actionable recommendations for budget management optimization. Ultimately, the findings are intended to improve resource allocation efficiency, enhance overall financial performance, and provide valuable references for policy makers and industry regulators. The effectiveness of budget control is highly contingent on the institutional environment and economic context in which it operates. Differences across countries in regulatory frameworks, corporate governance structures, and market uncertainty can significantly influence the practical efficacy of budget management tools. As a representative transitional economy, Russian enterprises face unique challenges in budget management, including institutional transitions, high market volatility, and heterogeneous governance structures. On one hand, firms in Russia generally rely on relatively strict internal budget control mechanisms to cope with external uncertainties and financing constraints. On the other hand, their corporate governance systems are still evolving, providing a distinctive institutional context for studying the relationship between budget control and financial performance. In this setting, examining Russian enterprises allows for an empirical investigation of how budget control functions under institutional and environmental constraints, thereby enriching management accounting theory with evidence from diverse national and economic contexts.

## Literature review

As an essential tool of management accounting, budget control plays a central role in resource allocation and the achievement of corporate objectives. Both domestic and international scholars have extensively explored its underlying mechanisms. Nguyen (2024) proposed that budget control effectively reduced operational uncertainty through planning and feedback mechanisms, thereby enhancing managerial control over resource allocation [6]. However, this study was limited by its sample composition, which focused primarily on traditional manufacturing industries and failed to capture the managerial variations present in emerging sectors. Zeng et al. (2023) pointed out that the balance between flexibility and constraint in the budgeting process directly determined its effectiveness; overly rigid budgeting practices suppressed corporate innovation vitality [7]. Nevertheless, their research relied solely on qualitative interviews and lacked systematic quantitative validation, which limited the generalizability of its conclusions. Financial performance, as a key indicator of corporate competitiveness and sustainability, had been widely studied in relation to internal governance, external environment, and accounting practices. Xu and Loang (2023) demonstrated that corporate governance structure had a significant impact on financial performance, particularly showing that improved internal control systems enhanced profitability [8]. However, their analysis gave insufficient attention to budget control and did not integrate it into a comprehensive performance-impact model. Kang et al. (2024), using cross-country empirical data, argued that external market conditions and industry characteristics accounted for performance disparities across firms. Yet their study did not further investigate the moderating role of budget management in these relationships [9]. In recent years, some scholars began to examine the interactive relationship between budget control and financial performance, emphasizing the role of budgeting in corporate value creation. Rahmah and Peter (2024) proposed that scientific budget management significantly improved financial performance by enhancing fund utilization efficiency. However, they did not account for potential moderating effects—such as firm size or industry differences—which might have introduced bias in the results [10]. Similarly, Makridou et al. (2024), based on empirical data, confirmed a positive relationship between budget control and financial performance but failed to incorporate mediation analysis, thereby overlooking indirect pathways such as management processes and incentive mechanisms [11].

From a theoretical perspective, the impact of budget control on financial performance is not a simple linear relationship but operates through a series of internal management mechanisms and contextual conditions [12]. Both agency theory and the resource-based view provide important theoretical support for understanding the pathways through which budget control affects performance. Agency theory suggests that in modern firms, information asymmetry and goal incongruence between owners and managers often generate agency costs. Budget control, as a formalized management control tool, helps mitigate opportunistic behavior by setting clear budget targets, regulating resource usage, and reinforcing execution and feedback mechanisms. In this process, the standardization of internal management processes and the improvement of resource allocation efficiency serve as key mediating mechanisms for the governance effects of budget control [13,14]. A well-designed budgeting system can standardize and increase transparency in internal processes, enhance cross-departmental coordination, and improve overall managerial control. Simultaneously, budget constraints and performance evaluation mechanisms guide resources toward high-efficiency, high-return business areas, boosting overall resource utilization. From the agency theory perspective, treating internal process optimization and resource allocation efficiency as mediators between budget control and financial performance has clear theoretical grounding. The resource-based view further emphasizes that a firm’s competitive advantage arises from its ability to effectively integrate and deploy key resources, rather than from resource endowments alone. In this framework, budget control is viewed as an institutional resource that indirectly shapes a firm’s value creation by influencing resource allocation and organizational operations. Resource allocation efficiency reflects the firm’s ability to transform limited resources into performance outputs, representing the core of “resource utilization capability.” Internal process optimization embodies organizational routines and managerial capabilities developed over time, forming a foundation for sustained competitive advantage [15]. Accordingly, from a resource-based perspective, budget control improves financial performance by enhancing resource allocation efficiency and optimizing internal management processes, demonstrating logical consistency in its mechanism.

Moreover, these theories also provide justification for the selection of moderating variables. Agency theory indicates that governance structure and firm size can influence the effectiveness of control mechanisms, with more robust governance facilitating effective implementation of budget control. The resource-based view and contingency perspective highlight that the performance effect of management tools depends on external conditions. Under high external uncertainty, budget control may face challenges such as implementation difficulty or reduced flexibility, potentially weakening its positive impact on financial performance. Therefore, including firm size, governance structure, and external environmental uncertainty as moderating variables helps reveal how the effectiveness of budget control varies across different contexts.

Overall, existing research has largely relied on qualitative analyses or simple regression models, lacking systematic quantitative validation and multi-dimensional testing. Moreover, few studies have incorporated budget control into a comprehensive analytical framework of financial performance, neglecting the mechanisms of mediating and moderating effects. To address these gaps, this study constructs a systematic model grounded in management accounting and financial performance theory, encompassing budget control, independent and dependent variables, as well as mediating and moderating variables. Building on descriptive statistics and correlation analysis, the study further employs multiple regression, moderation, and mediation effect tests, thereby establishing a more robust and integrated empirical framework.

## Research design

### Theoretical foundation and research framework

This study was primarily grounded in the following theoretical foundations (Table 1).

**Table 1:** Theoretical Foundations.

Theory	Analysis
Management Accounting Theory	Emphasized the use of tools such as budgeting, cost control, and performance evaluation to support strategic implementation and business decision-making. As a key component of management accounting, budget control served as a quantitative tool for resource allocation as an institutional arrangement for internal governance.
Agency Theory	In corporate management, information asymmetry and conflicts of interest commonly existed between shareholders (principals) and managers (agents). Budget control, by clarifying operational objectives, constraining agent behavior, and assessing performance, helped mitigate agency problems.
Resource-Based Theory	The competitive advantage of a firm originated from its rare and inimitable resources and the efficiency of their allocation. As an institutional resource, budget control could optimize fund allocation and enhance resource utilization efficiency, thereby fostering sustainable competitive advantage and improving financial performance.
Contingency Theory	The relationship between budget control and financial performance was not fixed but rather influenced by factors such as external environment, firm size, and governance structure [16]. Contingency theory emphasized that the effectiveness of management tools depended on their fit with contextual conditions.

Based on these theoretical perspectives, this study constructed a research framework on the relationship between budget control and financial performance. The framework included:

1. Independent Variable: Level of budget control, reflecting the degree of completeness in budgeting formulation, execution, and feedback mechanisms.
2. Dependent Variable: Financial performance, measured by indicators such as profitability and market value.
3. Mediating Variables: Internal management processes and resource allocation efficiency, representing the indirect mechanisms through which budget control affected financial performance.
4. Moderating Variables: Firm size, industrial environment, and governance structure, reflecting contextual differences in the relationship between budget control and financial performance.

From a logical perspective, this study first hypothesized that budget control had a direct positive effect on financial performance. Subsequently, it introduced mediating and moderating effects to explore how budget control indirectly influenced financial performance through improved management mechanisms and contextual adaptability. Finally, the study employed empirical data to test these hypotheses and provide a systematic explanation of the mechanisms underlying the effect of budget control.

### Research hypotheses

Grounded in the above theoretical foundations and framework, and considering both mediating and moderating pathways, the following hypotheses were proposed:

- H1: A higher level of budget control was associated with better financial performance [17].
- H2a: Resource allocation efficiency mediated the relationship between budget control and financial performance.
- H2b: Optimization of internal management processes mediated the relationship between budget control and financial performance [18,19].
- H3a: Firm size moderated the relationship between budget control and financial performance.
- H3b: Environmental uncertainty moderated the relationship between budget control and financial performance.
- H3c: Governance structure moderated the relationship between budget control and financial performance.

By integrating the direct, mediating, and moderating effects, the relationship between budget control and financial performance exhibited a combined mechanism of “direct influence–indirect influence–contextual variation” [20,21]. In other words, budget control directly affected financial performance, indirectly influenced it through mediating variables, and its strength and direction varied across different contexts.

### Model construction

Based on the theoretical foundations and research hypotheses presented earlier, this study constructed a research model to examine the relationship between budget control and financial performance. The overall model structure comprised three hierarchical paths:

1. Direct Effect Path: Budget control, as the independent variable, directly influenced corporate financial performance.
2. Mediating Effect Path: Budget control indirectly enhanced financial performance by improving resource allocation efficiency and optimizing internal management processes [22].
3. Moderating Effect Path: Contextual factors such as firm size, environmental uncertainty, and governance structure moderated the relationship between budget control and financial performance.

Accordingly, the proposed study model integrated both direct and indirect paths as well as moderating effects, aiming to comprehensively reveal the mechanisms through which budget control affected financial performance. The model’s rationality was reflected in its clear hierarchical structure and comprehensive mechanism design, which jointly captured direct, indirect, and contextual effects in a systematic manner. In addition, methodological diversity was ensured through the combined use of traditional regression analysis and mediation–moderation effect testing, which enhanced the robustness and explanatory power of the results. Furthermore, the model demonstrated strong practical operability by adopting a data collection approach that combined survey data with financial indicators, enabling empirical validation and offering managerial implications for enterprise practice.

### Experimental design

The experimental dataset used in this study was the Russian Financial Statements Database (RFSD), an open-access dataset available at https://github.com/irlcode/RFSD. The dataset covered unconsolidated firm-level financial statements of Russian companies from 2011 to 2023, including key financial documents such as balance sheets, income statements, and cash flow statements. This dataset was deemed suitable for the present research context. To test the proposed hypotheses, four categories of core variables were designed in accordance with management accounting and financial management theories, as shown in Table 2.

**Table 2:** Variable Design.

Dimension	Variable	Analysis
Independent Variable: Budget Control Level	Budget formulation rationality	Whether the budget was prepared based on historical data, market forecasts, and strategic objectives.
	Budget execution strictness	The degree of deviation between planned resource allocation and actual expenditures during budget implementation.
	Budget feedback timeliness	The soundness of corrective and improvement mechanisms during budget execution.
Dependent Variable: Financial Performance	Profitability indicators	Measured by return on assets (ROA) and return on equity (ROE).
	Market value indicators	Measured by Tobin’s Q to reflect market performance and investment value.
Mediating Variables	Resource allocation efficiency	Reflected whether financial, material, and human resources were allocated efficiently. Measured using a combination of questionnaire items and financial turnover ratios.
	Internal management process optimization	Measured whether budget management improved operational processes, including process standardization, information transparency, and execution efficiency.
Moderating Variables	Firm size	Measured by total assets or number of employees.
	Environmental uncertainty	Assessed through survey items measuring perceived market volatility, policy changes, and competitive intensity.
	Governance structure	Measured by board size and the proportion of independent directors.

To systematically test the research hypotheses, this study designed a structured questionnaire to quantify budget control, perceived financial performance, mediating variables, and moderating variables. The questionnaire consists of two parts: a basic information section and a core measurement section. The core measurement section includes 23 items, while the basic information section includes 5 items, resulting in a total of 28 questions. All measurement items are rated using a five-point Likert scale, where 1 indicates “strongly disagree” and 5 indicates “strongly agree.” A total of 500 questionnaires were distributed, and 477 valid responses were collected, yielding a response rate of 95.4%. The specific measurement items for each construct are listed in Appendix C. The scales were developed based on established constructs in management accounting, budget control, organizational process management, and contingency governance. They were further adapted to the context of budget management and governance in Russian enterprises. Budget control is measured across three dimensions: budget formulation rationality, budget execution strictness, and budget feedback timeliness. Resource allocation efficiency, internal process optimization, environmental uncertainty, and governance structure support are measured using their respective item groups. For reliability analysis, Cronbach’s alpha coefficients were calculated for all multi-item constructs. The results show that budget control, resource allocation efficiency, internal process optimization, environmental uncertainty, and perceived governance structure all have Cronbach’s alpha values above the commonly accepted threshold of 0.70. This indicates good internal consistency across constructs and satisfactory overall reliability of the questionnaire. For validity analysis, exploratory factor analysis and convergent validity tests were conducted. The Kaiser–Meyer–Olkin (KMO) measure and Bartlett’s test of sphericity indicate that the sample data are suitable for factor analysis. The extracted factor structure is consistent with the theoretical construct design. The standardized factor loadings of retained items fall within acceptable ranges. In addition, the average variance extracted and composite reliability of the main constructs meet standard criteria, indicating adequate convergent validity.

Since the regression analysis is conducted at the firm level, the questionnaire data were aggregated to construct firm-level composite variables. First, item scores within each dimension were averaged for each respondent to obtain first-order construct scores. Then, the scores for budget formulation, budget execution, and budget feedback were averaged to form the firm-level budget control variable used in the regression model. The same aggregation procedure was applied to resource allocation efficiency, internal process optimization, environmental uncertainty, and governance structure perception. For firms successfully matched with the RFSD database, if only one valid questionnaire was available, the respondent’s construct scores were directly used as firm-level values and matched with objective financial indicators from RFSD. This procedure ensures that the construction of composite variables is transparent and replicable. For financial performance, objective firm-level indicators from the RFSD database were used. These include return on total assets, return on equity, and Tobin’s Q. In the regression models, financial performance is treated as an objective outcome variable. It is clearly distinguished from perception-based variables derived from the questionnaire, which ensures consistency in data sources and measurement logic.

This study employed a questionnaire survey targeting middle and senior managers and combined it with firm-level secondary financial data from an open-access database for empirical analysis. Before completing the questionnaire, all respondents were clearly informed of the study’s purpose, the voluntary nature of participation, the scope of data usage, and the principle of anonymity. After reading the informed consent statement presented on the first page of the questionnaire, respondents chose whether to proceed and submit their responses. Submission of the questionnaire was regarded as provision of electronic informed consent. All participants in this study were adults with full legal capacity. The study did not involve minors, patients, or other vulnerable groups. During data collection, no personally identifiable information was recorded, such as names, identification numbers, or contact details. All responses were stored and analyzed in an anonymous form. The firm-level financial data used in this study were obtained from the RFSD, which is publicly accessible. The database contains only firm-level financial information and does not include any data that can identify individuals. This study is a non-invasive social science investigation. It does not involve biomedical experimentation, behavioral intervention, or the collection of sensitive personal data. All questionnaire data were collected anonymously and based on voluntary participation. According to commonly accepted ethical standards in social science and management research, such studies do not typically require formal approval from an Institutional Review Board (IRB) or an ethics committee. This study does not involve high-risk conditions that require additional written consent or special ethical safeguards. Therefore, the use of electronic informed consent is sufficient to meet ethical requirements.

This study employs a combination of survey data and secondary financial data for empirical analysis. To ensure consistency and comparability across different data sources, the selection of survey respondents was strictly limited to firms with continuous and complete financial records in the Russian Financial Statement Database (RFSD). Specifically, the survey was not randomly distributed to firms but targeted mid- to senior-level managers of sample companies in the RFSD database, such as CFOs, budget managers, or department heads.

To enhance the transparency and reproducibility of the data integration process between survey data and firm-level financial data, this study divides the matching procedure into four steps. First, firms were selected from the RFSD database covering the period from 2011 to 2023. Only firms with continuous financial records, complete core financial statements, and minimal missing values in key financial indicators were retained. Based on these criteria, 1,286 firms were identified as the initial sample pool. Second, questionnaires were distributed to middle and senior managers of the firms in the initial sample pool. Third, after the questionnaires were collected, each survey response was matched with RFSD firm-level financial records using three identifiers: firm name, registration number, and industry classification. When firm names contained abbreviations or minor spelling variations, the registration number was used as the primary identifier, and industry classification was applied for cross-verification to reduce the risk of mismatches. Fourth, sample cleaning was conducted. Questionnaires that could not be clearly matched to RFSD records were removed. Questionnaires with inconsistencies in key identifiers or core financial data were also excluded. Among the 477 valid responses, 39 were excluded due to matching failure or data inconsistency. This resulted in a final matched sample of 438 firms for subsequent regression analysis. In the final sample, variables such as budget control, resource allocation efficiency, internal process optimization, and environmental uncertainty were derived from the managerial survey data, reflecting internal management mechanisms and managerial perceptions. Financial performance variables, including return on total assets, return on equity, and Tobin’s Q, were obtained from objective financial indicators in the RFSD database. By matching subjective survey data with objective financial data at the firm level, this study reduces the risk of common method bias associated with single data sources and improves the internal consistency and explanatory power of the empirical analysis.

## Data evaluation and analysis

### Descriptive analysis

**Fig 1:**
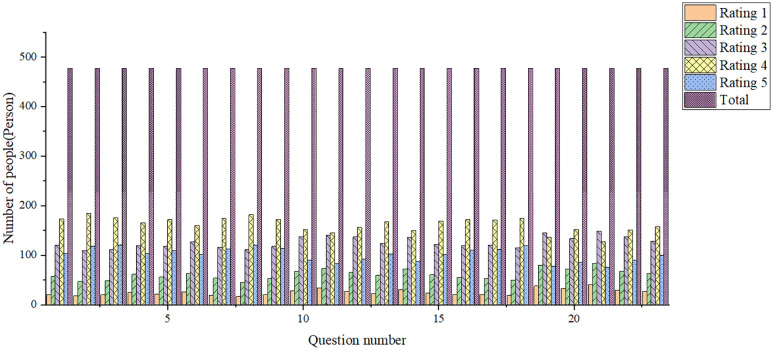
Descriptive Statistics ofCore Measures.

The descriptive statistics for the core measurement items are illustrated in Fig 1.

The descriptive statistics for the basic information section are summarized in Table 3.

**Table 3:** Basic Information Statistics.

Indicator	Category	Sample Size	Proportion
Ownership Type	State-owned	122	25.6%
	Private	244	51.2%
	Foreign/Joint Venture	111	23.2%
Industry Category	Manufacturing	188	39.4%
	Services	136	28.5%
	Technology/Information	95	19.9%
	Other	58	12.2%
Years of Establishment	≤5 years	94	19.7%
	6–10 years	141	29.6%
	11–20 years	163	34.2%
	>20 years	79	16.5%
Number of Employees	<100	112	23.5%
	100–499	173	36.3%
	500–999	108	22.6%
	≥1000	84	17.6%
Annual Revenue (CNY)	<50 million	105	22.0%
	50 million–200 million	148	31.0%
	200 million–1 billion	146	30.6%
	>1 billion	78	16.4%

Based on the results presented in Fig 1 and Table 3, private enterprises accounted for more than half of the sample, while state-owned and foreign/joint venture firms maintained a substantial presence. This indicates that the sample encompassed multiple ownership types, adequately reflecting the management practices of different types of firms. Regarding industry distribution, manufacturing firms represented the largest proportion, followed by service and technology/information industries, which aligns closely with current economic structural trends and enhances the practical representativeness of the sample. In terms of firm age, most enterprises were in the 6–20 year maturity stage, encompassing both growth-oriented firms and some long-established traditional firms. The distribution of employee size and annual revenue was relatively balanced, with small- and medium-sized enterprises comprising the majority, while large firms also accounted for a notable share, providing opportunities for subsequent heterogeneity analyses. Overall, the distribution of firm characteristics was reasonable, supporting the generalizability of the empirical study. Regarding the core measurement variables, the mean values of budget control ranged from 3.7 to 3.9, indicating that firms generally demonstrated a relatively high level of performance across budget formulation, execution, and feedback. Further observation revealed that the means for budget formulation and feedback were slightly higher than that for budget execution, suggesting that firms placed greater emphasis on early-stage scientific design and post-implementation improvement, while strict execution still showed some gaps. Financial performance had mean values concentrated between 3.5 and 3.6, slightly lower than budget control, indicating that most firms’ evaluations of profitability and market value were moderately high, with room for further improvement.

For the mediating variables, resource allocation efficiency had mean values of 3.6–3.7, reflecting that firms were able to improve resource utilization to some extent under the budget system, though variations remained noticeable. Internal management process optimization had slightly higher mean values of 3.8–3.9, demonstrating that the budget system was particularly effective in promoting process standardization and information transparency. Among the moderating variables, environmental uncertainty had mean values between 3.4 and 3.6, indicating that firms generally perceived a moderate level of market volatility and policy change pressures, while perceived governance structure had mean values of 3.6–3.7, suggesting a moderately high evaluation of governance arrangements and their positive support for budget implementation.

### Correlation analysis and multiple linear testing

**Fig 2:**
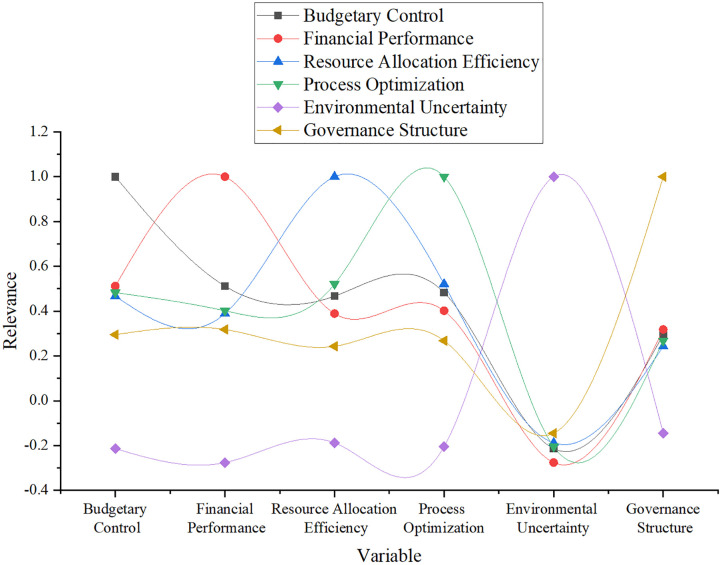
Correlation Analysis Results.

To further examine the relationships among the main variables and verify the assumptions underlying the research hypotheses, Pearson correlation coefficients were calculated for pairwise analysis. The results are presented in Fig 2.

As shown in Fig 2, budget control was significantly positively correlated with financial performance (r = 0.512, p < 0.01), providing preliminary support for Hypothesis H1. Additionally, budget control was significantly positively correlated with the mediating variables, resource allocation efficiency and internal process optimization, and financial performance was also significantly positively correlated with these mediators, laying the foundation for subsequent mediation analysis. Moreover, environmental uncertainty exhibited significant negative correlations with both budget control and financial performance, indicating that external fluctuations may weaken firms’ management effectiveness. Conversely, governance structure was positively correlated with both budget control and financial performance, suggesting that strong governance supports effective budget implementation. Based on the correlation analysis, multicollinearity among the main independent variables was further assessed using the variance inflation factor (VIF). Results are reported in Table 4.

**Table 4:** Multicollinearity Test Results.

Variable	Tolerance	VIF
Budget Preparation Rationality	0.641	1.560
Budget Execution Strictness	0.628	1.592
Budget Feedback Timeliness	0.615	1.626
Profitability Indicators	0.703	1.422
Market Value Indicators	0.689	1.451
Resource Allocation Efficiency	0.588	1.701
Internal Management Process Optimization	0.573	1.745
Firm Size	0.792	1.262
External Environmental Uncertainty	0.815	1.227
Governance Structure	0.801	1.248

As shown in Table 4, all VIF values were between 1 and 2, well below the commonly used threshold (VIF < 10), indicating that multicollinearity was not a concern, allowing the analysis to proceed to regression modeling.

### Regression analysis

To examine the direct effect of budget control on financial performance, this study employs multiple linear regression analysis. Given that the primary objective of this study is to examine the direct effect of budget control on corporate financial performance and its explanatory power, and that the final regression sample consists of firm-level observations obtained after questionnaire matching, a multiple linear regression approach was adopted. The questionnaire variables were collected from a one-time managerial survey rather than repeated observations over multiple years. Therefore, the dataset constitutes matched cross-sectional data at the firm level rather than balanced or unbalanced panel data. For this reason, panel data models were not employed. Although structural equation modeling is suitable for analyzing relationships among latent variables, this study focuses on the marginal explanatory power of budget control after controlling for firm characteristics. Accordingly, a hierarchical regression approach was adopted, as it better aligns with the research objective. A hierarchical regression approach was adopted, incrementally introducing key explanatory variables to assess the incremental explanatory power of budget control on financial performance after accounting for other factors. Specifically, financial performance serves as the dependent variable, with budget control variables as the main independent variables. A set of control variables is included to mitigate potential omitted variable bias. These controls—firm type, industry category, firm age, employee size, and operating revenue—are widely recognized in the literature as systematic determinants of financial performance. Financial performance is not represented by a single indicator but is operationalized using firm-level objective financial measures. Specifically, return on total assets, return on equity, and Tobin’s Q are used to capture corporate financial performance. These indicators are standardized prior to regression analysis to reduce the influence of differences in measurement scales. In the model specification, financial performance is constructed as a composite standardized outcome variable. This approach improves the consistency and comparability of the estimation results.

Based on this framework, the regression models are specified as follows:


$$\begin{array}{c} FP_{i}=\alpha_{\text{0}}+\alpha_{\text{1}}Nature_{i}+\alpha_{\text{2}}Industry_{i}+\alpha_{\text{3}}Age_{i}+\alpha_{\text{4}}Size_{i}+\alpha_{\text{5}}Revenue_{i}+\varepsilon_{i} \end{array}$$ (1)

$$\begin{array}{c} FP_{i}=\beta_{\text{0}}+\beta_{\text{1}}BC_{i}+\beta_{\text{2}}Nature_{i}+\beta_{\text{3}}Industry_{i}+\beta_{\text{4}}Age_{i}+\beta_{\text{5}}Size_{i}+\beta_{\text{6}}Revenue_{i}+\varepsilon_{i} \end{array}$$ (2)

To further improve the completeness of the model specification and examine the differential effects of various dimensions of budget control on financial performance, this study introduces the following extended model:


$$\begin{array}{c} FP_{i}=\gamma_{\text{0}}+\gamma_{\text{1}}BF_{i}+\gamma_{\text{2}}BE_{i}+\gamma_{\text{3}}FB_{i}+\gamma_{\text{4}}Nature_{i}+\gamma_{\text{5}}Industry_{i}+\gamma_{\text{6}}Age_{i}+\gamma_{\text{7}}Size_{i}+\gamma_{\text{8}}Revenue_{i}+\varepsilon_{i} \end{array}$$ (3)

In this model, $BF_{i},\;{\;\gamma }_{\text{2}}BE_{i},\;\gamma_{\text{3}}FB_{i}$ represent budget formulation rationality, budget execution strictness, and budget feedback timeliness, respectively. Where $FP_{i}$ represents the financial performance of firm *i*, $BC_{i}$ denotes the level of budget control, $Nature_{i}$, $Industry_{i}$, $Age_{i}$, $Size_{i}$, and $Revenue_{i}$ correspond to firm type, industry category, firm age, number of employees, and operating revenue, respectively, and $\varepsilon_{i}$ is the random error term. The regression results are presented in Table 5.

**Table 5:** Regression Analysis Results.

Variable	Model 1 (Control Variables Only)	Model 2 (Including Budget Control)
Ownership Type	0.082 (0.067)	0.051 (0.062)
Industry	0.091 (0.059)	0.063 (0.054)
Firm Age	0.073 (0.041)	0.046 (0.038)
Employee Size	0.116 (0.052) *	0.084 (0.047)
Annual Revenue	0.142 (0.048) **	0.097 (0.043) *
Budget Control	—	0.428 (0.051) ***
Constant	2.417 (0.212) ***	1.926 (0.198) ***
R²	0.183	0.361
Adjusted R²	0.162	0.344
F-value	8.621***	15.734***

*Note: Standard errors in parentheses; *p < 0.05, **p < 0.01, ***p < 0.001

Model 1 includes only the control variables and captures the explanatory power of basic firm characteristics on financial performance. Model 1 includes only control variables and is used to assess the explanatory power of basic firm characteristics on financial performance. The R² is 0.183, indicating that firm nature, industry category, firm age, employee size, and revenue explain approximately 18.3% of the variation in financial performance. In Model 2, the budget control variable is introduced. Its regression coefficient is 0.428 and is significantly positive at the 1% level. This result indicates that, after controlling for firm characteristics, budget control has a significant positive effect on financial performance, thereby supporting Hypothesis H1. Meanwhile, the R² increases from 0.183 to 0.361, and the adjusted R² increases from 0.162 to 0.344. This improvement suggests that the inclusion of budget control substantially enhances the explanatory power of the model. The incremental explanatory effect is not only statistically significant but also meaningful from a managerial perspective. To ensure the robustness of the estimation results, heteroskedasticity tests were conducted. The results indicate that no severe heteroskedasticity is present. In addition, robust standard errors were used in parameter estimation to mitigate the potential impact of heteroskedasticity on statistical inference. This approach improves the reliability and rigor of the regression results.

This incremental effect is both statistically and practically meaningful. Additionally, employee size and operating revenue show positive effects in some specifications, suggesting that larger firms with higher revenues tend to achieve better financial performance. However, their influence is noticeably weaker than that of budget control, further highlighting the central role of budget control in shaping firm performance. Despite the RFSD database covering firm-level financial information from 2011 to 2023, this study did not construct a firm–year panel regression model. The primary reason is that the key explanatory variables, such as budget control, were derived from a one-time questionnaire survey. These variables cannot provide continuous observations that correspond to financial indicators on an annual basis. If a panel model were applied directly, it would introduce temporal inconsistency between managerial variables and financial variables, thereby weakening the interpretability of the estimation results. Therefore, this study adopted a cross-sectional regression framework based on the matched firm-level sample, ensuring consistency between variable construction and model specification.

### Mediation and moderation analysis

Mediation effects were tested using Baron & Kenny’s three-step regression procedure combined with Bootstrap resampling (5,000 repetitions). Results are presented in Table 6.

**Table 6:** Mediation Effect Test Results.

Path	Coefficient	t-value	Significance
Budget Control → Financial Performance	0.428	8.39	***
Budget Control → Resource Allocation Efficiency	0.467	7.56	***
Budget Control → Internal Process Optimization	0.483	7.84	***
Resource Allocation Efficiency → Financial Performance	0.219	4.12	***
Internal Process Optimization → Financial Performance	0.238	4.33	***
Indirect Effect	0.178	—	95% CI = [0.112, 0.249]

*Note: ***p < 0.001

The results indicate that the direct effect of budget control on financial performance was significant (β = 0.428, t = 8.39, p < 0.001), while budget control also indirectly affected financial performance through resource allocation efficiency (β = 0.219, t = 4.12, p < 0.001) and internal process optimization (β = 0.238, t = 4.33, p < 0.001). The Bootstrap 95% confidence interval for the indirect effect [0.112, 0.249] did not include zero, confirming the significance of the mediation effect. The indirect effect accounted for 29.4% of the total effect, indicating that budget control enhances financial performance both directly and indirectly through management mechanisms. To test Hypothesis H3, interaction terms (e.g., Budget Control × Firm Size) were included in the regression model to examine the moderating effects of contextual variables. The results are reported in Table 7.

**Table 7:** Moderation Effect Test Results.

Variable	Firm Size	Environmental Uncertainty	Governance Structure
Budget Control → Financial Performance	0.401***	0.422***	0.416***
Interaction Term (BC × Moderator)	0.127**	−0.153**	0.139**
ΔR²	+0.032	+0.028	+0.031

*Note: **p < 0.01, **p < 0.001

The results in Table 7 indicate that the interaction coefficient for firm size was 0.127 (p < 0.01), suggesting that the effect of budget control on financial performance is stronger in larger firms. Specifically, in firms with greater size, the marginal impact of budget control on financial performance increases by approximately 12.7%. For environmental uncertainty, the interaction coefficient was −0.153 (p < 0.01), indicating that when market fluctuations and policy conditions are unstable, the positive effect of budget control is weakened, with explanatory power reduced by approximately 15.3%. The interaction coefficient for governance structure was 0.139 (p < 0.01), showing that higher governance quality strengthens the relationship between budget control and financial performance, increasing the positive effect by roughly 13.9%. Furthermore, including these three moderators increased the model R² by 2.8%–3.2%, further confirming the robustness of the moderation effects. These findings provide empirical support for Hypotheses H3a, H3b, and H3c.

## Discussion

Budget control has a significant positive impact on financial performance. This conclusion aligns with existing literature, indicating that scientific budget preparation, strict execution, and timely feedback can enhance corporate resource allocation efficiency and improve overall operational outcomes. This suggests that the budgeting system is not merely a formalistic tool, but an essential mechanism for achieving strategic implementation and operational objectives. Contrary to some studies that argue the effect of budget execution is limited, the results of this study demonstrate that its impact on financial performance is substantive. The mediation analysis revealed that budget control indirectly affects financial performance through improvements in resource allocation efficiency and internal management processes. In other words, the budgeting system has a direct effect and promotes financial performance by optimizing resource utilization, standardizing processes, and enhancing managerial transparency. This finding confirms the multifaceted value of budget control as a management accounting tool and supplements existing research by clarifying its underlying mechanisms, indicating that improvements in financial performance rely on the synergistic effect of institutionalized management and internal operational efficiency. The moderation analysis indicated that different contextual factors significantly influence the effectiveness of budget control. Firm size and governance structure reinforce the effect of the budgeting system, highlighting the importance of implementation capability and organizational support. Conversely, environmental uncertainty diminishes the effectiveness of budget control, suggesting that budget management does not operate with equal efficiency under all conditions. These conclusions support the core tenets of contingency theory, which posits that the effectiveness of management practices depends on their alignment with environmental conditions.

The findings of this study are derived from Russian firms and should be interpreted in the context of the country’s specific institutional and economic environment. Russian enterprises generally face high external uncertainty, significant resource constraints, and heterogeneous corporate governance structures, which make budget control particularly critical for resource allocation and internal process standardization. Therefore, the observed positive effect of budget control on financial performance reflects, to some extent, the practical need for firms in transitional economies to rely on formal management mechanisms to cope with environmental complexity. By contrast, in economies with more stable institutional frameworks and mature market mechanisms, the marginal impact and operational pathways of budget control may differ. Accordingly, the results of this study should not be generalized as universally applicable principles, but rather interpreted as empirical evidence relevant to Russia and countries with similar institutional contexts. Future research could employ cross-country comparisons or multi-region samples to examine how the mechanisms of budget control vary under different institutional and economic conditions, thereby enhancing the external validity and generalizability of the findings.

In addition, the theoretical contribution of this study lies not only in confirming a significant positive relationship between budget control and financial performance, but also in revealing the underlying mechanisms and contextual boundaries through which this relationship operates. Existing literature has largely focused on whether budget control improves firm performance, while relatively limited attention has been paid to how this effect is generated and under what conditions it becomes stronger or weaker. By introducing resource allocation efficiency and internal process optimization as mediating variables, this study shows that budget control does not affect financial performance in a direct or isolated manner. Instead, it improves internal resource allocation, enhances process standardization, and increases information transparency, which in turn translate into better performance outcomes. In this sense, the study extends the literature from simple correlation testing to mechanism-based explanation, thereby deepening the theoretical interpretation of budget control effects in management accounting research. Furthermore, by incorporating firm size, environmental uncertainty, and governance structure as moderating variables, this study demonstrates that the performance effect of budget control is not uniform across organizational contexts. Instead, it exhibits clear contingency characteristics. This finding suggests that budget control should not be viewed as a static or universally effective management tool. Rather, it should be understood as a dynamic management accounting mechanism embedded within governance structures, organizational scale conditions, and external environmental constraints. Compared with studies focusing solely on direct effects, the integrated “mediation–moderation” framework developed in this study addresses both why budget control is effective and under what conditions it becomes more effective. This provides a more comprehensive analytical perspective for the management accounting literature. In addition, by decomposing budget control into budget formulation, budget execution, and budget feedback, and integrating these dimensions into a unified framework, this study moves beyond treating budget control as a single construct. This perspective further strengthens the understanding of the closed-loop logic of budget control. Budget control is reflected in the rationality of ex ante planning, and in the enforceability of in-process execution and the corrective function of ex post feedback. Accordingly, this study contributes to shifting management accounting research from evaluating the effectiveness of budgeting tools toward analyzing the operational mechanisms of budgeting systems. Finally, in the context of Russian firms as a transitional economy, this study further shows that the mechanism of budget control is shaped by internal management practices and by institutional volatility, governance heterogeneity, and organizational maturity. Therefore, this study does not merely replicate existing findings in a new regional context. Instead, it extends the boundary conditions of budget control theory under a more uncertain institutional environment. This provides more context-sensitive empirical evidence for the application of management accounting theory in transitional economies.

Although this study carefully matched questionnaire data with RFSD financial data using firm name, registration number, and industry information, and excluded 39 observations due to incomplete or inconsistent matching among 477 valid responses, the integration process may still introduce matching bias and survival bias. First, firms included in the final matched sample of 438 are more likely to have more standardized disclosure practices, more complete financial records, and more stable operating conditions. As a result, the final sample may overrepresent firms with higher managerial standardization, better data completeness, and stronger going-concern capacity. Second, the RFSD screening process retained only firms with continuous financial records and complete core statements from 2011 to 2023. This implies that firms with missing financial data, high volatility, or market exit were excluded from the analysis, which may introduce survival bias. Therefore, the empirical results mainly reflect the relationship between budget control and financial performance among firms with relatively complete financial records and successful data matching. The external validity for firms with weak disclosure quality, short survival periods, or unstable operations is relatively limited. While this approach improves measurement consistency and matching accuracy, future research could further examine potential selection bias by comparing matched and unmatched firms, incorporating exit samples, or integrating additional external databases.

## Conclusion

Based on 477 valid samples, this study constructed and empirically tested a model of the relationship between budget control and financial performance. Through descriptive analysis, correlation testing, regression analysis, and mediation and moderation effect tests, the study demonstrates that budget control has a significant positive effect on financial performance. Scientific budget preparation, strict execution, and timely feedback effectively enhance resource allocation efficiency and reduce uncertainty in internal management, thereby improving profitability and market performance. Moreover, budget control also indirectly influences financial performance through mediation pathways. Resource allocation efficiency and internal management process optimization play a critical role between budget control and financial performance, indicating that the budgeting system enhances performance not only at the goal-setting level but also through improvements in managerial mechanisms and operational processes. Additionally, the effect of budget control is moderated by contextual factors. Firm size and governance structure strengthen the positive impact of budget control on financial performance, whereas environmental uncertainty weakens its effectiveness.

Although this study provides theoretical and practical insights, several limitations exist. First, the measurement of financial performance partly relied on a combination of financial indicators and survey-based perceptions, which may introduce subjective bias. Future research could incorporate third-party databases or longitudinal financial data to enhance objectivity and accuracy. Second, this study primarily employed regression analysis and mediation/moderation tests. While effective in revealing relationships between variables, these methods represent a static analysis. Future studies could adopt structural equation modeling, multilevel modeling, or panel data techniques to further improve the explanatory power of the model.

## Supporting information

S1 File Appendix A provides the anonymized questionnaire response dataset and related data files. Appendix B provides detailed information on variable coding and the reproducible analytical procedures. Appendix C presents the survey instrument titled “Questionnaire on Corporate Budget Control and Financial Performance.”
(ZIP)

S1 Data Supplementary data, variable definitions, and analytical procedures related to research reproducibility are available in the above appendices. (XLSX)
