# Supplementary material for: Construction and empirical analysis of a quantitative model on the relationship between budget control and financial performance in management accounting—Evidence from Russian enterprises
Source: PLoS One. 2026 Jul 15;21(7):e0337863. doi: 10.1371/journal.pone.0337863 (PMC13372177; doi:10.1371/journal.pone.0337863)
Supplement: S1 File — Appendix B provides detailed information on variable coding and the reproducible analytical procedures. Appendix C presents the survey instrument titled “Questionnaire on Corporate Budget Control and Financial Performance.” (ZIP) [file pone.0337863.s001.zip › the supplementary material/Appendix B.docx]

"""

Reproducible Analysis Pipeline: RFSD + Survey Dataset (Anonymized)

Author: <Your Name>

Purpose:

- Load and merge RFSD financial data with anonymized survey responses

- Compute construct scores

- Run descriptive statistics, VIF, multiple linear regression

- Run mediation (bootstrap) and moderation (interaction) analyses

Notes:

- This script assumes you provide TWO anonymized files:

1) survey_with_basic_info.xlsx (or .csv) containing Respondent_ID, Q1..Q23, plus firm characteristics

2) rfsd_financials.csv containing firm-level financial metrics matched by Firm_ID (or a consistent key)

- Replace file paths and column names to match your actual anonymized dataset.

"""

from __future__ import annotations

import os

import numpy as np

import pandas as pd

import statsmodels.api as sm

from statsmodels.stats.outliers_influence import variance_inflation_factor

# -----------------------------

# 1) Configuration

# -----------------------------

SURVEY_FILE = "survey_with_basic_info_randomized.xlsx" # Appendix 2 (anonymized respondent-level data)

SURVEY_SHEET_CORE = "Core_Questionnaire"

SURVEY_SHEET_BASIC = "Basic_Information"

RFSD_FILE = "rfsd_financials.csv" # Public or processed RFSD extract

MERGE_KEY_SURVEY = "Firm_ID" # You MUST provide this in the anonymized survey dataset

MERGE_KEY_RFSD = "Firm_ID" # Consistent firm key in the RFSD extract

RANDOM_SEED = 42

BOOTSTRAP_N = 5000

np.random.seed(RANDOM_SEED)

# -----------------------------

# 2) Helper Functions

# -----------------------------

def safe_zscore(s: pd.Series) -> pd.Series:

"""Z-score with safe handling for constant series."""

std = s.std(ddof=0)

if std == 0 or np.isnan(std):

return (s - s.mean()) * 0.0

return (s - s.mean()) / std

def add_constant(df: pd.DataFrame) -> pd.DataFrame:

"""Add intercept column for statsmodels."""

return sm.add_constant(df, has_constant="add")

def compute_vif(X: pd.DataFrame) -> pd.DataFrame:

"""Compute VIF table for predictors."""

Xc = add_constant(X).copy()

vif_rows = []

cols = list(Xc.columns)

for i, c in enumerate(cols):

if c == "const":

continue

vif = variance_inflation_factor(Xc.values, i)

tol = 1.0 / vif if vif != 0 else np.nan

vif_rows.append({"Variable": c, "Tolerance": tol, "VIF": vif})

return pd.DataFrame(vif_rows).sort_values("VIF", ascending=False)

def ols_fit(y: pd.Series, X: pd.DataFrame):

"""Fit OLS with robust standard errors (HC3) to be conservative."""

model = sm.OLS(y, add_constant(X))

res = model.fit(cov_type="HC3")

return res

def bootstrap_indirect_effect(

data: pd.DataFrame,

x: str,

m_list: list[str],

y: str,

covariates: list[str],

n_boot: int = 5000,

) -> dict:

"""

Parallel mediation bootstrap:

X -> M1, M2 ... -> Y

Returns bootstrap CI for total indirect effect.

"""

effects = []

n = len(data)

for _ in range(n_boot):

sample = data.sample(n=n, replace=True)

# a-paths: X -> Mk

a = {}

for m in m_list:

res_a = ols_fit(sample[m], sample[[x] + covariates])

a[m] = res_a.params.get(x, np.nan)

# b-paths: Mk -> Y controlling for X and covariates

res_b = ols_fit(sample[y], sample[[x] + m_list + covariates])

b = {m: res_b.params.get(m, np.nan) for m in m_list}

# total indirect = sum(a_k * b_k)

ind = np.nansum([a[m] * b[m] for m in m_list])

effects.append(ind)

effects = np.array(effects)

ci_low, ci_high = np.percentile(effects, [2.5, 97.5])

return {

"bootstrap_n": n_boot,

"indirect_mean": float(np.mean(effects)),

"ci_95_low": float(ci_low),

"ci_95_high": float(ci_high),

}

# -----------------------------

# 3) Load Survey Data (Appendix 2)

# -----------------------------

if not os.path.exists(SURVEY_FILE):

raise FileNotFoundError(f"Survey file not found: {SURVEY_FILE}")

core = pd.read_excel(SURVEY_FILE, sheet_name=SURVEY_SHEET_CORE)

basic = pd.read_excel(SURVEY_FILE, sheet_name=SURVEY_SHEET_BASIC)

# Merge core + basic by Respondent_ID

survey = core.merge(basic, on="Respondent_ID", how="inner")

# IMPORTANT: You must add a firm-level matching key into the anonymized dataset

# Example: survey["Firm_ID"] = <your anonymized firm id>

if MERGE_KEY_SURVEY not in survey.columns:

raise KeyError(

f"Missing merge key '{MERGE_KEY_SURVEY}' in survey. "

"Please include an anonymized Firm_ID in Appendix 2 for firm-level matching."

)

# -----------------------------

# 4) Construct Variables from Questionnaire Items (example mapping)

# -----------------------------

# Budget control (BC): compilation, execution, feedback

BC_compilation = ["Q1", "Q2", "Q3"]

BC_execution = ["Q4", "Q5", "Q6"]

BC_feedback = ["Q7", "Q8", "Q9"]

# Mediators (example):

RA_efficiency = ["Q10", "Q11", "Q12", "Q13"] # resource allocation efficiency

IP_process = ["Q14", "Q15", "Q16", "Q17"] # internal process optimization

# Moderators (example):

EU_uncertainty = ["Q18", "Q19", "Q20"] # environmental uncertainty

# Governance structure is often objective (board size, independent ratio).

# If you have perception items, map them here; otherwise you will use RFSD/hand-collected governance data.

GS_governance = ["Q21", "Q22", "Q23"] # governance perception (if survey-based)

# Create composite scores (mean aggregation)

survey["BC_Compilation"] = survey[BC_compilation].mean(axis=1)

survey["BC_Execution"] = survey[BC_execution].mean(axis=1)

survey["BC_Feedback"] = survey[BC_feedback].mean(axis=1)

survey["Budget_Control"] = survey[["BC_Compilation", "BC_Execution", "BC_Feedback"]].mean(axis=1)

survey["Resource_Allocation"] = survey[RA_efficiency].mean(axis=1)

survey["Internal_Process"] = survey[IP_process].mean(axis=1)

survey["Env_Uncertainty"] = survey[EU_uncertainty].mean(axis=1)

survey["Governance_Structure"] = survey[GS_governance].mean(axis=1)

# Optional: encode categorical controls (basic info)

# Keep them as dummies for regression

controls_cat = ["Ownership", "Industry", "Firm_Age", "Firm_Size", "Annual_Revenue"]

for c in controls_cat:

if c not in survey.columns:

raise KeyError(f"Missing control column in survey basic info: {c}")

survey_dummies = pd.get_dummies(survey, columns=controls_cat, drop_first=True)

# -----------------------------

# 5) Load RFSD Financial Data

# -----------------------------

if not os.path.exists(RFSD_FILE):

raise FileNotFoundError(f"RFSD file not found: {RFSD_FILE}")

rfsd = pd.read_csv(RFSD_FILE)

if MERGE_KEY_RFSD not in rfsd.columns:

raise KeyError(f"Missing merge key '{MERGE_KEY_RFSD}' in RFSD extract.")

# Example financial performance variables (you should adjust to your actual extract):

# ROA, ROE, TobinsQ

required_fin_cols = ["ROA", "ROE", "TobinsQ"]

for col in required_fin_cols:

if col not in rfsd.columns:

raise KeyError(f"Missing required financial column in RFSD extract: {col}")

# Aggregate multiple years to a single firm-level value if needed (example: mean 2011-2023)

# If your RFSD extract is already firm-level, skip this.

if "Year" in rfsd.columns:

fin = rfsd.groupby(MERGE_KEY_RFSD)[required_fin_cols].mean().reset_index()

else:

fin = rfsd[[MERGE_KEY_RFSD] + required_fin_cols].copy()

# Create a standardized financial performance index (optional)

fin["FP_Index"] = safe_zscore(fin["ROA"]) + safe_zscore(fin["ROE"]) + safe_zscore(fin["TobinsQ"])

fin["FP_Index"] = fin["FP_Index"] / 3.0

# -----------------------------

# 6) Merge Survey + RFSD by Firm_ID

# -----------------------------

data = survey_dummies.merge(fin, left_on=MERGE_KEY_SURVEY, right_on=MERGE_KEY_RFSD, how="inner")

print(f"[INFO] Matched sample size (firms/respondents): {len(data)}")

# -----------------------------

# 7) Descriptive Statistics

# -----------------------------

desc_cols = [

"Budget_Control", "Resource_Allocation", "Internal_Process",

"Env_Uncertainty", "Governance_Structure", "FP_Index",

]

print("\n[DESCRIPTIVE STATISTICS]")

print(data[desc_cols].describe().T[["mean", "std", "min", "max"]])

# -----------------------------

# 8) Multicollinearity (VIF)

# -----------------------------

# Predictors for the direct-effect regression

control_dummy_cols = [c for c in data.columns if any(c.startswith(k + "_") for k in controls_cat)]

X_vif = data[["Budget_Control"] + control_dummy_cols].copy()

vif_table = compute_vif(X_vif)

print("\n[VIF TABLE]")

print(vif_table)

# -----------------------------

# 9) Multiple Linear Regression (Hierarchical Models)

# -----------------------------

# Model 1: controls only

y = data["FP_Index"]

X1 = data[control_dummy_cols]

res1 = ols_fit(y, X1)

# Model 2: controls + budget control

X2 = data[["Budget_Control"] + control_dummy_cols]

res2 = ols_fit(y, X2)

print("\n[REGRESSION RESULTS: MODEL 1 (Controls Only)]")

print(res1.summary())

print("\n[REGRESSION RESULTS: MODEL 2 (Add Budget_Control)]")

print(res2.summary())

print("\n[R-SQUARED CHANGE]")

print(f"R^2 Model 1: {res1.rsquared:.3f}")

print(f"R^2 Model 2: {res2.rsquared:.3f}")

print(f"ΔR^2: {(res2.rsquared - res1.rsquared):.3f}")

# -----------------------------

# 10) Mediation Analysis (Bootstrap)

# -----------------------------

# X = Budget_Control; Mediators = Resource_Allocation, Internal_Process; Y = FP_Index

mediation_result = bootstrap_indirect_effect(

data=data,

x="Budget_Control",

m_list=["Resource_Allocation", "Internal_Process"],

y="FP_Index",

covariates=control_dummy_cols,

n_boot=BOOTSTRAP_N,

)

print("\n[BOOTSTRAP MEDIATION: TOTAL INDIRECT EFFECT]")

print(mediation_result)

# -----------------------------

# 11) Moderation Analysis (Interaction Terms)

# -----------------------------

# Example moderators: Firm size (if numeric) OR Env_Uncertainty, Governance_Structure

# Here we use Env_Uncertainty and Governance_Structure as moderators.

data["BC_x_EnvU"] = data["Budget_Control"] * data["Env_Uncertainty"]

data["BC_x_Gov"] = data["Budget_Control"] * data["Governance_Structure"]

# Model 3: add interaction with Env_Uncertainty

X3 = data[["Budget_Control", "Env_Uncertainty", "BC_x_EnvU"] + control_dummy_cols]

res3 = ols_fit(y, X3)

# Model 4: add interaction with Governance_Structure

X4 = data[["Budget_Control", "Governance_Structure", "BC_x_Gov"] + control_dummy_cols]

res4 = ols_fit(y, X4)

print("\n[MODERATION RESULTS: MODEL 3 (Env_Uncertainty Interaction)]")

print(res3.summary())

print("\n[MODERATION RESULTS: MODEL 4 (Governance Interaction)]")

print(res4.summary())

print("\n[DONE] The pipeline finished successfully.")

print("Please archive the anonymized dataset files and this script as Supplementary Materials.")
