# Supplementary material for: Construction and empirical analysis of a quantitative model on the relationship between budget control and financial performance in management accounting—Evidence from Russian enterprises
Source: PLoS One. 2026 Jul 15;21(7):e0337863. doi: 10.1371/journal.pone.0337863 (PMC13372177; doi:10.1371/journal.pone.0337863)
Supplement: S1 File — Appendix B provides detailed information on variable coding and the reproducible analytical procedures. Appendix C presents the survey instrument titled “Questionnaire on Corporate Budget Control and Financial Performance.” (ZIP) [file pone.0337863.s001.zip › the supplementary material/Appendix C.docx]

**Questionnaire on Corporate Budget Control and Financial Performance**

Thank you for taking the time to participate in this academic survey. This questionnaire aims to examine the relationship between corporate budget control and financial performance, as well as the underlying mechanisms. The results will be used solely for academic research and will not involve any commercial purposes.

The questionnaire is anonymous. All responses will be kept strictly confidential and reported only in aggregated statistical form. Please answer the questions based on your company’s actual situation and your own judgment.

This questionnaire consists of two parts:

- **Part I:** Basic information about the company.
- **Part II:** Core measurement items related to budget management and performance.

There are no right or wrong answers. Please respond honestly. Thank you for your support and cooperation.

**Part I: Basic Information (5 items)**

Please select the option that best describes your company:

**B1. Nature of the enterprise**
☐ State-owned enterprise
☐ Private enterprise
☐ Foreign-funded / joint venture

**B2. Industry sector**
☐ Manufacturing
☐ Services
☐ Technology / information industry
☐ Other (please specify): ________

**B3. Years since establishment**
☐ Less than 5 years
☐ 6–10 years
☐ 11–20 years
☐ More than 20 years

**B4. Number of employees**
☐ Fewer than 100
☐ 100–499
☐ 500–999
☐ 1,000 or more

**B5. Annual revenue**
☐ Less than 50 million RMB
☐ 50–200 million RMB
☐ 200 million–1 billion RMB
☐ More than 1 billion RMB

**Part II: Core Measurement Items (23 items)**

**Instructions:**
All items are measured using a five-point Likert scale:
1 = Strongly disagree
2 = Disagree
3 = Neutral
4 = Agree
5 = Strongly agree

Please select the option that best reflects your company’s actual situation.

**(1) Budget Control — Budget Formulation (BC1–BC3)**

**BC1** The company fully considers historical operational data when preparing budgets.
**BC2** Market forecasts and industry trends are systematically incorporated into the budgeting process.
**BC3** Budget targets are highly aligned with the company’s overall development strategy.

**(2) Budget Control — Budget Execution (BC4–BC6)**

**BC4** The company strictly controls expenditures during budget execution.
**BC5** Deviations between actual and budgeted expenditures are generally within a controllable range.
**BC6** The company effectively constrains unplanned expenditures during budget execution.

**(3) Budget Control — Feedback and Timeliness (BC7–BC9)**

**BC7** The company promptly analyzes and responds to budget deviations.
**BC8** Budget execution results are used for subsequent adjustments and improvements.
**BC9** The company has established a well-developed budget evaluation and feedback mechanism.

**(4) Resource Allocation Efficiency (RA1–RA4)**

**RA1** Financial resource allocation effectively supports core business development.
**RA2** Human resource allocation is generally appropriate.
**RA3** There is a high level of alignment between resource input and output.
**RA4** Budget management helps improve overall resource utilization efficiency.

**(5) Internal Process Optimization (IP1–IP4)**

**IP1** Budget management promotes the standardization of internal processes.
**IP2** Budget systems enhance internal information transparency.
**IP3** Budget management improves coordination among departments.
**IP4** Budget management enhances overall execution efficiency.

**(6) External Environmental Uncertainty (EU1–EU3)**

**EU1** The market environment in which the company operates changes frequently.
**EU2** Policy adjustments have a significant impact on business decisions.
**EU3** Industry competition introduces substantial uncertainty into operations.

**(7) Perceived Financial Performance (FP1–FP3)**

**FP1** The company has demonstrated strong profitability in recent years.
**FP2** The company’s asset utilization efficiency is relatively high within the industry.
**FP3** The company’s market performance and investment value are generally satisfactory.

Thank you again for your participation and support.

If you are interested in the final results of this study, you are welcome to follow the related academic publications.
We wish you success in your work and continued professional advancement.
